# Preparation and Characterization of Dextran Coated Iron Oxide Nanoparticles Thin Layers

**DOI:** 10.3390/polym13142351

**Published:** 2021-07-18

**Authors:** Gabriel Predoi, Carmen Steluta Ciobanu, Simona Liliana Iconaru, Daniela Predoi, Dragana Biliana Dreghici, Andreea Groza, Florica Barbuceanu, Carmen Cimpeanu, Monica-Luminita Badea, Stefania-Felicia Barbuceanu, Ciprian Florin Furnaris, Cristian Belu, Liliana Ghegoiu, Mariana Stefania Raita

**Affiliations:** 1 Faculty of Veterinary Medicine, University of Agronomic Sciences and Veterinary Medicine of Bucharest, 105 Splaiul Independentei, Sector 5, 050097 Bucharest, Romania; flori.barbuceanu@yahoo.com (F.B.); ffurnaris@gmail.com (C.F.F.); cristbelu@yahoo.com (C.B.); 2 Multifunctional Materials and Structures Laboratory, National Institute of Materials Physics, Atomistilor Street, No. 405A, P.O. Box MG 07, 077125 Magurele, Romania; simonaiconaru@gmail.com (S.L.I.); dpredoi@gmail.com (D.P.); monibadea78@gmail.com (M.-L.B.); lilibadea82@yahoo.com (L.G.); 3 Low Temperature Plasma Laboratory, National Institute for Laser, Plasma and Radiation Physics, 409 Atomistilor Street, P.O. Box MG 36, 077125 Magurele, Romania; dragana.dreghici@inflpr.ro (D.B.D.); andreeagroza75@gmail.com (A.G.); 4 Institute for Diagnosis and Animal Health, 63 Staicovici D. Nicolae, Street, 50557 Bucharest, Romania; 5 Faculty of Land Reclamation and Environmental Engineering, University of Agronomic Sciences and Veterinary Medicine of Bucharest, 59 Marasti Blvd, Sector 1, 011464 Bucharest, Romania; carmencimpeanu@yahoo.com; 6 Faculty of Horticulture, University of Agronomic Sciences and Veterinary Medicine, 59 Marasti Blvd., 011464 Bucharest, Romania; 7 Organic Chemistry Department, Faculty of Pharmacy, University of Medicine and Pharmacy, Traian Vuia Street 6, 020956 Bucharest, Romania; stefaniafelicia_barbuceanu@yahoo.com

**Keywords:** iron oxide, dextran, thin film, glass substrate, polymers, morphology, biocompatibility, glow discharge optical emission spectrometry, MTT assay

## Abstract

In the present study, we report the synthesis of a dextran coated iron oxide nanoparticles (DIO-NPs) thin layer on glass substrate by an adapted method. The surface morphology of the obtained samples was analyzed by Scanning Electron Microscopy (SEM), Atomic Force Microscopy (AFM), optical, and metallographic microscopies. In addition, the distribution of the chemical elements into the DIO-NPs thin layer was analyzed by Glow Discharge Optical Emission Spectrometry (GDOES). Furthermore, the chemical bonds formed between the dextran and iron oxide nanoparticles was investigated by Fourier Transform Infrared Spectroscopy (FTIR). Additionally, the HepG2 viability incubated with the DIO-NPs layers was evaluated at different time intervals using MTT (3-(4, 5-dimethylthiazol-2-yl)-2,5-diphenyltetrazolium bromide) assay. The goal of this study was to obtain a DIO-NPs thin layer which could be used as a coating for medical devices such as microfluidic channel, microchips, and catheter. The results of the surface morphology investigations conducted on DIO-NPs thin layer suggests the presence of a continuous and homogeneous layer. In addition, the GDOES results indicate the presence of C, H, Fe, and O signal intensities characteristic to the DIO-NPs layers. The presence in the IR spectra of the Fe-CO metal carbonyl vibration bonds prove that the linkage between iron oxide nanoparticles and dextran take place through carbon–oxygen bonds. The cytotoxicity assays highlighted that HepG2 cells morphology did not show any noticeable modifications after being incubated with DIO-NPs layers. In addition, the MTT assay suggested that the DIO-NPs layers did not present any toxic effects towards HEpG2 cells.

## 1. Introduction

Recently, among the most studied biomaterials due to their special magnetic and biological properties, iron oxide nanoparticles (especially magnetite (Fe_3_O_4_) and maghemite (γ-Fe_2_O_3_)) stand out [1,2]. One of the well-known applications of superparamagnetic iron oxide nanoparticles (SPION) is their use as a contrast agent in Magnetic Resonance Imaging (MRI) to visualize liver damage [3]. Previous studies [1] have shown that their unique properties such as high surface area to volume ratios and superparamagnetism (exhibited by particles with a diameter below 30 nm) [4,5] make them suitable for various applications. Therefore, due to their capability to be easily magnetized when they are exposed to the action of an external magnetic field, SPION could be used in various biomedical applications such as: drug and gene delivery [6,7] chelation therapy [8], hyperthermia [9,10], tissue repair [11,12], etc.

The iron oxide nanoparticle’s parameters such as shape, size, and properties could be controlled by the synthesis method [1]. SPION can be obtained by various methods of synthesis such as sol-gel, coprecipitation, thermal decomposition, hydrothermal method, etc. [5,13]. Co-precipitation synthesis is among the most used because it is a cheap and efficient method that allows the easy obtaining of superparamagnetic iron oxide nanoparticles [13]. In general, to prevent the agglomeration of nanoparticles and to improve their colloidal stability and biological properties, they are incorporated into polymers (chitosan, dextran, poly(vinyl alcohol)—PVA, gelatin, polyvinylpyrrolidone (PVP), etc.) [6,13,14,15,16]. Dextran, (H(C_6_H_10_O_5_)xOH), is one of the most widely used natural polysaccharides used as a coating for SPION [1]. In addition, dextran has antithrombotic and anticoagulant properties, it is nontoxic, and exhibits a good bioaffinity [1,17,18,19]. Therefore, the iron oxide–dextran composite (thin films and nanoparticles) are of great interest for biomedical applications, due to their superior biological properties.

A study conducted by A.K. Hauser et al. [20] highlighted that the stability in the PBS (Phosphate buffered saline solution) medium of dextran coated iron oxide nanoparticle is influenced by the amount of dextran used in synthesis. In addition, it was noticed that the synthesis method of dextran coated iron oxide nanoparticles greatly affects the physic-chemical properties of the samples [20]. Therefore, currently iron oxide–dextran compounds (crosslinked iron oxides (CLIO)) are being studied by a large number of researchers around the world, and the results obtained so far are encouraging, indicating that this type of compound could be used as a contrast agent in MRI [3,21]. In general, iron oxide layers can be obtained by various deposition methods (for example: DC-pulsed magnetron, electrodeposition, pulsed laser deposition, chemical vapor deposition, chemical spray pyrolysis technique, etc.) [22]. On the other hand, for the deposition of layers based on iron oxide / polymer, methods such as Matrix Assisted Pulsed Laser Evaporation (MAPLE), dip coating, spin coating, etc. could be used [23,24]. Encouraging results have been obtained by using SPION as a coating for medical devices due to the inhibition of microbial colonization induced by the presence of superparamagnetic iron oxide nanoparticles [25]. Thus, it is of real interest to obtain thin layers of magnetic materials. Previous studies conducted by Prodan and co-workers showed that the antimicrobial activity of iron oxide nanoparticles is influenced on the one hand by the iron concentration and on the other hand by the microbial growth state [26]. Moreover, a good biocompatibility with HepG2 cell line of dextran coated maghemite thin films was noticed [27]. Our previous studies reported the obtaining and characterization of iron oxide–dextran thin films on various substrates by various methods [23,24,27,28]. However, it is not yet fully understood how iron oxide nanoparticles interact with dextran and, on the other hand, further studies are needed to understand how the morphology of the layers influences the biological properties of the dextran coated iron oxides nanoparticles’ (DIO-NPs) thin layer. Therefore, on the one hand, iron oxide nanoparticles coated with dextran could be used as a contrast agent, drug carrier, etc. [29], while dextran-coated iron oxide layers could be used to cover prosthetic devices, medical surfaces, etc. [30]. The goal of this study was to obtain a DIO-NPs thin layer which could be used as a coating for medical devices/surfaces.

The novelty of this complex study consists of conducting, for the first time, a study regarding the surface morphology of the DIO-NPs layers for biomedical applications. For this purpose, four microscopy techniques were used (optical, metallographic, SEM, and AFM microscopy) which provided complete information on the surface morphology.

In this context, the main purpose of this study was to investigate the morphology and biocompatible properties of a dextran coated iron oxides nanoparticles (DIO-NPs) thin layer prepared by an adapted method. The morphology of the obtained thin films was analyzed using optical, metallographic examinations, scanning electron microscopy (SEM), and atomic force microscopy (AFM). In addition, Glow Discharge Optical Emission Spectrometry (GDOES) was used in order to study the distribution of the chemical elements into the bulk layer. Information regarding the vibrational properties of a dextran coated iron oxides nanoparticles (DIO-NPs) thin layer were obtained by Fourier-transform infrared spectroscopy (FTIR). Furthermore, the HepG2 viability incubated with the DIO-NPs layers was evaluated at 24, 48, and 72 h using the standard colorimetric MTT (3-(4, 5-dimethylthiazol-2-yl)-2,5-diphenyltetrazolium bromide) assay.

## 2. Materials and Methods

### 2.1. Materials

In order to develop the dextran coated iron oxide nanoparticles (DIO-NPs) thin layer, the starting materials such as dextran, H(C_6_H_10_O_5_)xOH, (Dextran from *Leuconostoc mesenteroides*, MW~40,000, Sigma, Darmstad, Germany), ferric chloride hexahidrate (FeCl_3_·6H_2_O, 97%, Sigma Aldrich, Darmstad, Germany), ferrous chloride tetrahydrate (FeCl_2_·4H_2_O, ≥99%, Sigma Aldrich, Darmstad, Germany), natrium hydroxide (NaOH, ≥97.0%, pellets, Sigma Aldrich, Darmstad, Germany), and hydrochloric acid (HCl solution, 37%, Merck) were purchased. In the synthesis of DIO-NPs and for the rinsing of clusters, we used deionized water. The glass substrate was purchased from Solaronix (Aubonne, Switzerland)

### 2.2. Thin Layer of DIO-NPs Synthesis

The suspension synthesis and layer preparation were based on an adapted method presented previously [23,31,32]. Ferrous chloride tetrahydrate and chloride hexahydrate were mixed at room temperature and added drop by drop into the dextran solution (20% *w/v*). The synthesis parameters (adjusting pH, ionic strength, temperature), including the time and rate of adding the base, were carefully monitored and optimized. In the recent study on preparation and characterization of highly stable iron oxide nanoparticles for magnetic resonance imaging [33], it was shown that the slow formation of nanoparticle seeds was followed by a faster formation of cores and a slow formation of stabilized shells. According to Massart studies on preparation of aqueous magnetic liquids in alkaline and acidic media [34], the increase of pH from 4 to 11 of the FeCl_2_/FeCl_3_ precursor solution by addition of the NaOH induces the co-precipitation of iron oxide particles. The most important factor of this synthesis was the iron concentration. The Fe^2+^/Fe^3+^ ratio was 0.5. According to previous studies [35,36,37], the particles obtained with the Fe^2+^/Fe^3+^ ratio between 0.4 and 0.6 are the most effective in biomedical applications. The particles (DIO-NPs) were washed several times to remove the residues. The obtained particles (DIO-NPs) were homogeneous in size and composition according to previous studies [30]. Moreover, previous studies on size-tunable synthesis of stable superparamagnetic iron oxide nanoparticles for potential biomedical applications [38] revealed that the dextran was used as a surfactant, enabling obtained particles to be used directly for biological applications without further surface functionalization. According to past studies [39], at room temperature, the concentration of iron salts is about 0.1 mol/L in terms of magnetite. The resulting suspension obtained was used to realize the DIO-NPs layers. Before the deposition process, the glass substrate was cleaned with ethanol in an ultrasonic bath for 12 min. Using a syringe, 0.2 mL of DIO-NPs suspension was dripped onto the glass substrate (1 × 1 cm^2^). The glass substrate was rotated during deposition at 1000 rpm for 40 s. The procedure was repeated 5 times. Immediately after coating, the layer was dried in a nitrogen atmosphere for 1 h at 70 °C. The resulting layer was treated at 100 °C under vacuum for 1 h.

### 2.3. Characterization of DIO-NPs Thin Layer

#### 2.3.1. Optical Microscopy

Optical microscopy was used in order to investigate the surface morphology of the DIO-NPs layers. Thus, for this purpose, a binocular optical microscope with an attached camera from Micros Austria (1.3 MP, Micros Austria, Wien, Austria) was used. The images were recorded at the 40X magnification using Microvisible software. All of the measurements were performed at room temperature. The 3D representation of optical microscopy images of the DIO-NPs layers surface was obtained using ImageJ software [40].

#### 2.3.2. Metallographic Microscopy

An inversed trinocular metallographic microscope OX.2153-PLM, (Euromex, Arnhem, The Netherlands) equipped with an CMEX digital camera (1.3 MP) was used in order to obtain additional information on the morphology of DIO-NPs thin layer. The metallographic images were captured using ImageFocusAlpha software at 20X magnification in ambient conditions. In addition, a 3D representation of 2D metallographic microscopy images of the DIO-NPs layers surface was obtained using ImageJ software [40].

#### 2.3.3. Scanning Electron Microscopy (SEM)

The surface morphology of the DIO-NPs layers and suspension has been investigated by scanning electron microscopy (SEM) with the aid of an Apreo S ThermoFisher Scanning Electron Microscope. Firstly, a drop of DIO-NPs suspension was placed on a double-sided adhesive carbon tape and dried in a vacuum, and then analyzed by SEM. The working voltage and pressure were 10 kV and 10^−3^ Pa. The 2D SEM recorded images have been processed by using the ImageJ software [40]. A 3D surface plot analysis of DIO-NP layers has also been performed. From the 2D image of the DIO-NP layer acquired with 100,000X magnification, the nanoparticles size distribution has been calculated. In addition, 800 particles were measured to establish the mean particle size distribution (D_SEM_).

#### 2.3.4. Atomic Force Microscopy (AFM)

It is well known that atomic force microscopy is a powerful technique that provides information about the morphology of the samples. Therefore, atomic force microscopy (AFM) was used in order to investigate the morphology of a DIO-NPs thin layer using an NT-MDT NTEGRA Probe Nano Laboratory instrument (NT-MDT, Moscow, Russia). For this purpose, a silicon NT-MDT NSG01 cantilever was used (NT-MDT, Moscow, Russia) coated with a 35 nm gold layer in non-contact mode. The instrument was set to acquire scans of 10 × 10 µm^2^ and 2.5 × 2.5 µm^2^ in a non-contact mode with a point number of 512 × 512 and a frequency of 0.4 Hz. The scanning data used in our study were the image of surface topography (signal Height).The data were acquired on a surface area of 10 × 10 µm^2^ in atmospheric conditions and at an ambient temperature of 25 ± 1 °C, and data processing was performed with the aid of Gwyddion 2.55 software (Department of Nanometrology, Czech Metrology Institute, Brno, Czech Republic) [41]. The roughness parameters of the 2D surface topography obtained by AFM were also determined using the dedicated “Calculate roughness parameters” of the Gwyddion software. The roughness parameters were determined from the entire 10 × 10 µm^2^ 2D AFM topography. In addition, the grain size distribution was determined using the statistical analysis of the AFM images. For the determination of the grain size, at least six AFM topographies of 2.5 × 2.5 µm^2^ and 10 × 10 µm^2^ were analyzed. The proportion of the grain area was calculated using the following equation:

(1) $$P_{i}=0.25\frac{d_{i}^{2}\pi n_{i}}{S}\;$$

where *d_i_* represents the diameter of a given grain, *S* stands for the area of all grains in the analyzed image, and *n_i_* corresponds to the number of grains of a given size. The distribution plots were fitted using the Gaussian function, and the grain size was determined at the values corresponding to the maxima of the functions.

Before the visualization under microscopes, the samples were dedusted using a compressed air gun.

#### 2.3.5. Fourier Transform Infrared Spectroscopy (FT-IR)

For study of the chemical bonds formed between the dextran and iron oxide nanoparticles, IR spectra analysis of the DIO-NPs layers has been performed. The spectra were acquired in the 400–4000 cm^−1^ wavenumber range in an attenuated total reflection (ATR) mode by using an SP 100 Perkin Elmer FTIR spectrometer (Waltham, MS, USA) with 4 cm^−1^ resolution. The spectra have been acquired in transmittance mode by placing the samples on the diamond-ZnSe crystal plate of the ATR unit inserted into the spectrometer and analyzed by using the Spectrum software version 6.3.5. The spectrum of the DIO-NPs layer was recorded after 32 scans.

#### 2.3.6. Glow Discharge Optical Emission Spectrometry (GDOES)

By Glow Discharge Optical Emission Spectrometry (GDOES), the distribution of the chemical elements into the layer bulk was analyzed. A GD Profiler (Horiba Company, Longjumeau, France) has been used. The procedure and the working parameters were: 650 Pa, 35 W RF power impulse mode at 1 kHz, and a duty cycle of 0.25.

#### 2.3.7. Cell Viability Assay

The biocompatibility of the DIO-NPs layers was investigated using the human hepatoma immortal cell line (HepG2). The HepG2 cell line was derived from the liver tissue of a 15-year-old Caucasian male having a well-differentiated hepatocellular carcinoma and nowadays is the most commonly studied liver cell line due to the advantages of availability and secretion of human proteins [42]. The cytotoxicity of the DIO-NPs against HepG2 cells was assessed using a method previously described in [27]. For the viability assays, the cells were grown in an environment having 5% CO_2_ at a temperature of 37 °C, as monolayers, and were seeded at a density of 2.5 × 10^5^ cells/mL and incubated with DIO-NPs layers’ different time intervals (24, 48, and 72 h). The HepG2 viability incubated with the DIO-NPs layers was evaluated at 24, 48, and 72 h, using the standard colorimetric MTT (3-(4, 5-dimethylthiazol-2-yl)-2,5-diphenyltetrazolium bromide) assay. The absorbance of the culture medium was quantified with a TECAN spectrophotometer at 595 nm. The data were quantified as previously detailed in [43].

#### 2.3.8. Statistical Analysis

The in vitro experiments were performed in triplicate. The data were presented as mean ± standard deviation (SD). In addition, the statistical analysis was done using two-sample *t*-tests. Only values of *p* ≤ 0.05 were considered statistically significant.

## 3. Results

**Figure 1:**
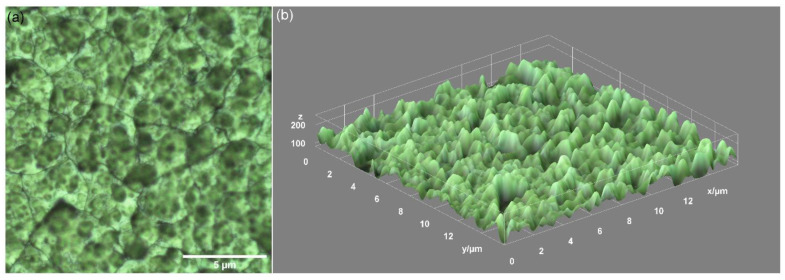
Optical image of DIO-NPs layers acquired with 40X objective (**a**) and 3D representation of the surface of the DIO-NPs layers (**b**).

By optical microscopy, the polymeric surface texture of the DIO-NPs layers deposited on the glass substrate is revealed at the micron scale. The optical image (40X magnification) of the coating presented in Figure 1a seems to be homogenous, indicating a uniform distribution of iron nanoparticles into dextran. By using the ImageJ software, the optical image of the layer has been processed. Figure 1b presents the 3D representation of the optical image of the DIO-NPs layer’s surface.

**Figure 2:**
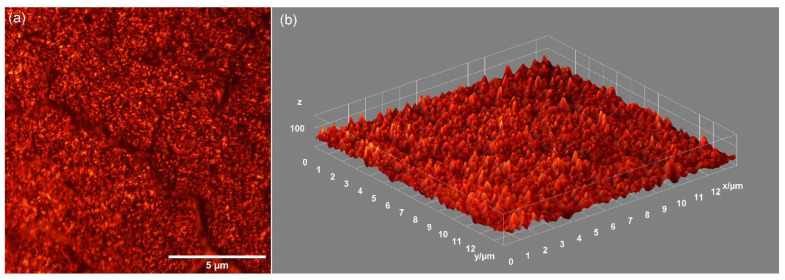
Metallographic microcopy image (20X magnification) of the DIO-NPs layers surface (**a**) and 3D representation of the surface of the DIO-NPs layers (**b**).

In addition, metallographic microscopy was used to obtain complementary information regarding the surface morphology of the DIO-NPs layers deposited on the glass substrate. The 2D metallographic image of DIO-NPs layers is presented in Figure 2a and the 3D representation of the metallographic image of DIO-NPs layers is also presented in Figure 2b. For this purpose, the 20X objective of the metallographic microscope was used in order to obtain a 2D image of the surface of DIO-NPs layers. For the obtaining of 3D representations of the surface of DIO-NPs layers, ImageJ software [40] was used. Thus, the results presented in Figure 2 suggest that the surface of the studied layers is continuous and homogeneous without noticing the presence of discontinuities or cracks.

**Figure 3:**
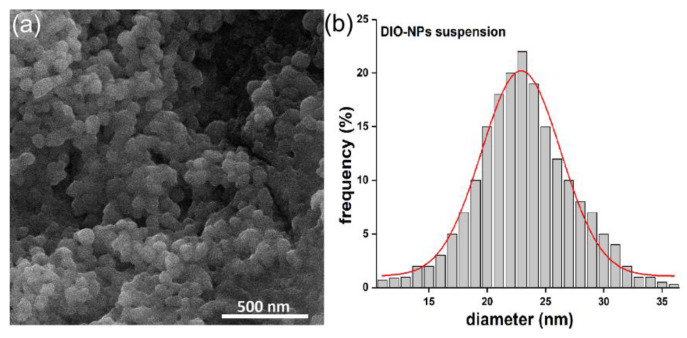
SEM image of DIO-NPs suspension (**a**) and particle size distribution (**b**).

The SEM micrograph along with particle size distribution of the DIO-NPs suspension are presented in Figure 3. The results of SEM studies conducted on the DIO-NPs suspension revealed that the nanoparticles possess a polyhedral morphology. In addition, in the obtained histogram, it could be observed that the medium particle diameter (D_SEM_) is 23 ± 3 nm (Figure 3b).

**Figure 4:**
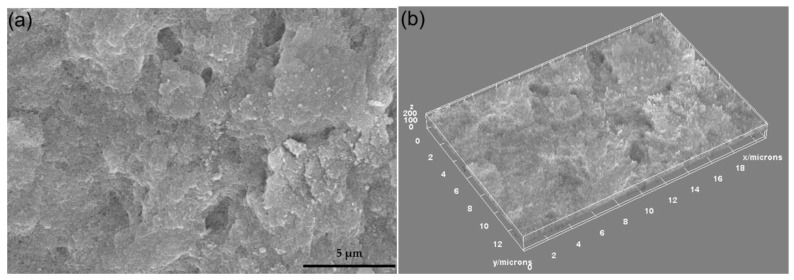
SEM image of DIO-NPs layers at 10,000× magnification (**a**) and 3D surface plot of SEM image of DIO-NPs layers at 10,000× magnification (**b**).

**Figure 5:**
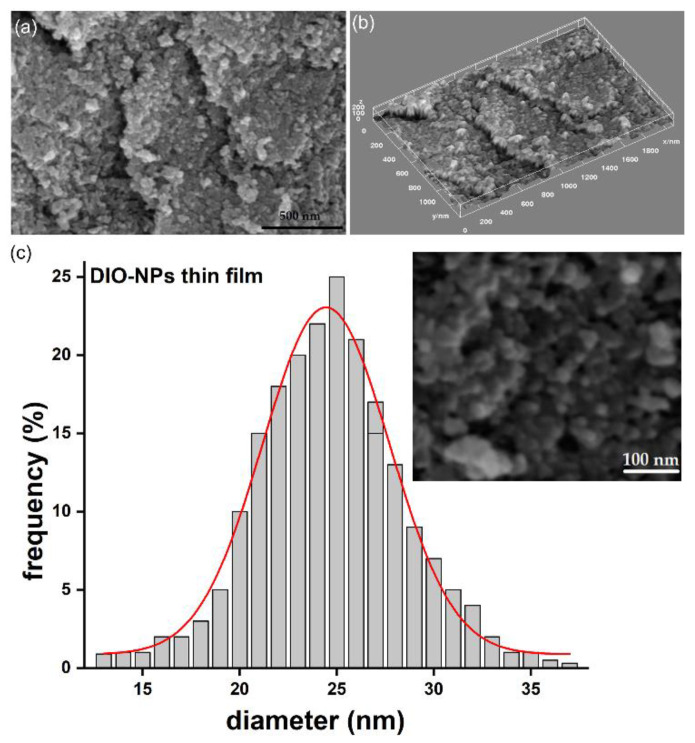
SEM image of DIO-NPs layers at 10,000× magnification (**a**), 3D surface plot of SEM image of DIO-NPs layers at 10,000× magnification (**b**) and size distribution of particles from SEM image-inset (**c**).

The analysis of DIO-NP layers by SEM at two microscope magnifications, namely 10,000× (Figure 4a) and 100,000× (Figure 5a), allowed the disclosure of the DIO-NPs layer surfaces with high accuracy.

On the micron scale, the SEM image from Figure 4a revealed the cobblestone—like structure of the layers. Such surface morphologies are characteristics to dextran layers [26]. The 3D representation of SEM image is presented in Figure 4b and give us the first hint about the embedding of the iron oxide nanoparticles into dextran.

The presence of the iron nanoparticles in dextran is uncovered at 100,000× magnification (Figure 5a), where their uniform and homogenous distribution can be observed, without affecting the cobblestone-like structures. Figure 5b exhibit the 3D plot of the SEM image of the DIO-NP layers. In addition, the particle size distribution of dextran coated iron oxide nanoparticle has been obtained (Figure 5c).

The analyses of the surface morphologies of the layers at different magnifications by SEM technique allowed the disclosure of micron-to nm structure of DIO-NP layers. Therefore, the distribution pattern of iron oxide nanoparticles embedded into dextran has been accurately revealed at 100,000× magnification of the SEM apparatus. The mean nanoparticle size is about 25 nm ± 3 nm (Figure 5c). On the micron analysis scale, the surface morphologies of the DIO-NP layers look similar to those observed by optical microscopy (Figure 1a).

**Figure 6:**
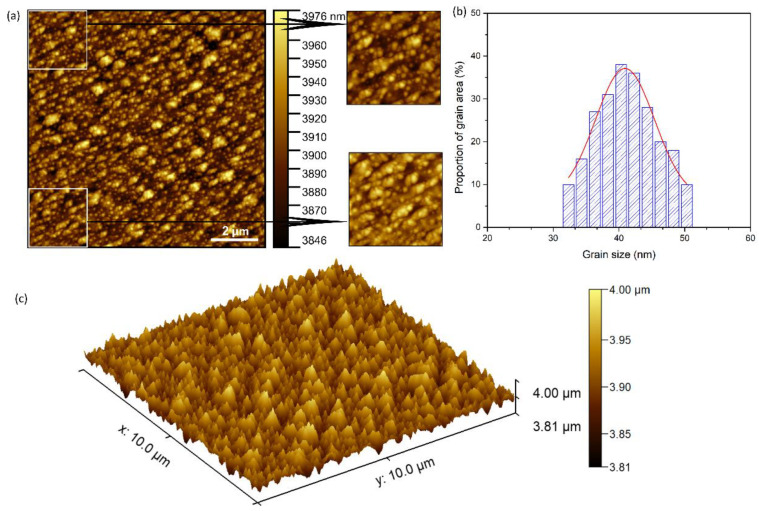
Atomic force microscopy (AFM) topography image of the DIO-NPs layers surface presented in 2D surface (size 10 × 10 µm^2^) and enlarged areas (size 2.5 × 2.5 µm^2^) (**a**), grain size distribution (**b**) and 3D representation of the surface topography of DIO-NPs layers (**c**).

In addition, Atomic Force Microscopy was used to study the surface of the DIO-NPs layers deposited on the glass substrate. The results of AFM studies are depicted in Figure 6. The AFM 2D micrograph of the DIO-NPs layers surface is shown in Figure 6a and the 3D representation of the DIO-NPs layers surface is presented in Figure 6b. Both the 2D AFM micrograph and the 3D representation of the surface of the DIO-NPs layers suggest the formation of a continuous and homogeneous layer. The roughness parameters (R_q_ and R_a_) of DIO-NPs layers surface estimated from AFM topography were 23.6738 nm and 19.1050 nm. These results suggest that the surface topography is homogenous and does not present a significant roughness. Moreover, in the AFM images (Figure 6), there is no evidence of the presence of cracks or fissures on the surface of DIO-NPs layers. In addition, the grain size distribution of the DIO-NPs was also determined using six 2D AFM topographies of 10 × 10 µm^2^ and 2.5 × 2.5 µm^2^. A representative 10 × 10 µm^2^ is presented in Figure 6a. Moreover, two of the enlarged 2.5 × 2.5 µm^2^ AFM sections used for the grain analysis and the grain size plot are also presented in Figure 6b. The sample preparation and the data processing were done using adapted methods of previously suggested methodology [44,45,46,47,48]. Furthermore, the 3D representation of the 10 × 10 AFM micrograph is also depicted in Figure 6c.

Complementary information about the grain size distribution of the DIO-NPs coatings is also determined from the optical microscopy, metallographic microscopy, and scanning electron microcopy. The results are presented in Table 1. The grain size distribution of the optical and metallographic microscopy images was determined using ImageJ software [40], and the results are presented as mean ± SD. For this purpose, at least three separate sections from the 2D images were analyzed using the “Particle Analyzer” function of the software. The grain size distribution from SEM images was determined by measuring the diameter of the particles and plotting the histogram, and the size distribution from AFM images was determined as previously described by Zubar et al. [47]. The size distribution obtained from the four complementary techniques were in the range of 25–97.5 nm and suggested that the coatings are comprised of a uniform deposited layer of nanometric particles and aggregates of nanometric particles. The particle agglomeration could be attributed to the presence of dextran coating of the iron oxide particles.

Furthermore, additional information regarding the roughness of the DIO-NPs coatings was also obtained using the data from the four complementary techniques (SEM, AFM, optical and metallographic microscopy). The data collected from the SEM, optical, and metallographic microscopy were used to determine the roughness parameters (R_a_ and R_q_). The results are presented in Table 2. The roughness parameters were determined using ImageJ software [40]. On the other hand, the AFM roughness parameters were calculated using Gwyddion software [41]. The data suggested that the values of the roughness average (R_a_) and root mean square (RMS) roughness (R_q_) obtained from the four complementary techniques were in the range of 19–114 nm in the case of (R_a_) and in the interval of 23–104.5 nm for (R_q_). The difference in the results obtained in our study regarding the roughness parameters determination by various techniques could be attributed to the limitation of the instruments in acquiring the images and also to their quality. Nonetheless, the values for the roughness parameters obtained from the four complementary techniques were no higher than 104.5 in the case of R_q_ and lower than 114.4 for the R_a_, which suggested a homogenous and uniform surface with a moderate to minimum roughness.

Therefore, analyzing the results obtained by the four complementary techniques (SEM, AFM, optical and metallographic microscopy), we can say that the morphology of the surface DIO-NPs layers is continuous and homogeneous without noticing the presence of discontinuities.

**Figure 7:**
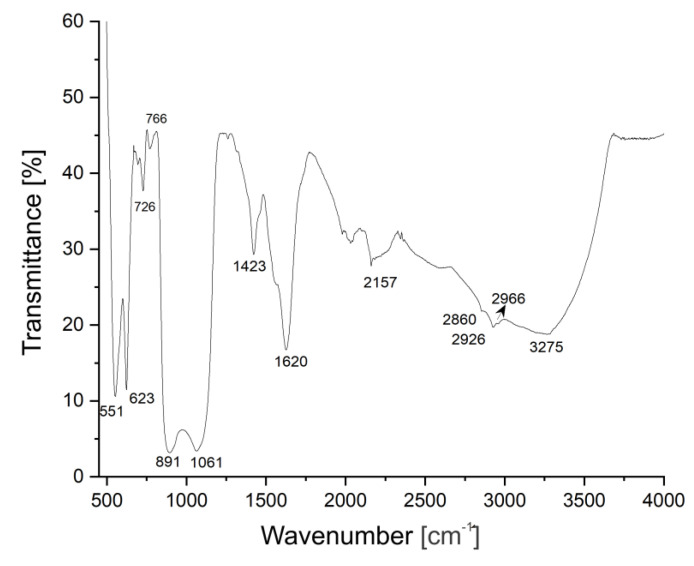
FTIR spectrum of DIO-NPs layers.

The FTIR spectrum of DIO-NPs layers deposited on the glass substrate is presented in Figure 7. The IR bands characteristic to Fe-O bonds, usually manifested in a 550–650 cm^−1^ spectral range [24], appear at 551 respectively 623 cm^−1^. The IR bands from 726, 891, and 1061 cm^−1^ are characteristic to the structure of dextran [24]. The peak intensity positioned at 766 cm^−1^ is characteristic to a1, 3 of the glycoside unit in dextran [49]. The stronger bands from 1061 cm^−1^ can be attributed to the C-O-C stretching mode in the ring [50], while the 891 cm^−1^ peak belongs to α-glucopyranose ring deformation modes of the C-O bonds [51]. The IR bands from 1423, 1620 cm^−1^ are attributed to C-H asymmetric bend and H-O-H vibrations, respectively [24,49,50,51,52]. The IR bands from 2860, 2926, and 2966 cm^−1^ are specific to symmetric/asymmetric stretch C-H vibrations in the methyl group [51]. The bands from 3275 cm^−1^ are assigned to O-H vibrations [51].

The strong IR band from 2157 cm^−1^ is specific to Fe-CO metal carbonyl vibration bonds [53]. Their presence in the FTIR spectrum of DIO-NPs layers proves that the linkage between iron oxide nanoparticles and dextran takes place through carbon–oxygen bonds.

The distribution of iron oxide nanoparticles into the dextran layer was also evaluated from the depth profiles of the C, H, Fe, and O elements, by using the GDOES techniques. In our previous work, we described in detail the working principle of this technique [42].

**Figure 8:**
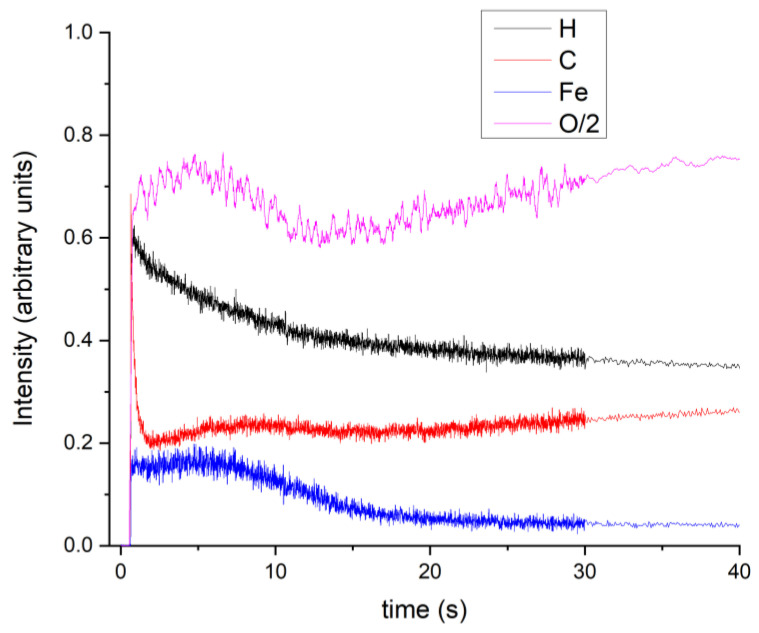
GDOES depth profile of the DIO-NPs layers.

Figure 8 presents the time dependence of the acquired C, H, Fe, and O signal intensities characteristic to the DIO-NPs layers. The depth profile of O has the highest intensity, as it is included both in the chemical structure of dextran and of the iron oxide nanoparticles. The simultaneous humps observed on the depth profile signal of O, C, and Fe suggest the embedding of iron oxide nanoparticles into dextran. Moreover, the O, Fe, and C signal profiles indicate that the iron oxides are linked to dextran via carbon–oxygen bonds.

By the GDOES technique, the temporal evolution of the recorded signals expresses the distribution of the elements contained in a layer from its surface up to the substrate interface. In Figure 8, the interface between the DIO-NPs layers and substrate is marked by the decrease of Fe depth profile. The smoothing of the C, H, O, and Fe depth profile signals after 30 s indicates the ending of the measurements.

Therefore, the GDOES results are in good agreement with the results obtained by FTIR studies on the DIO-NPs layers.

**Figure 9:**
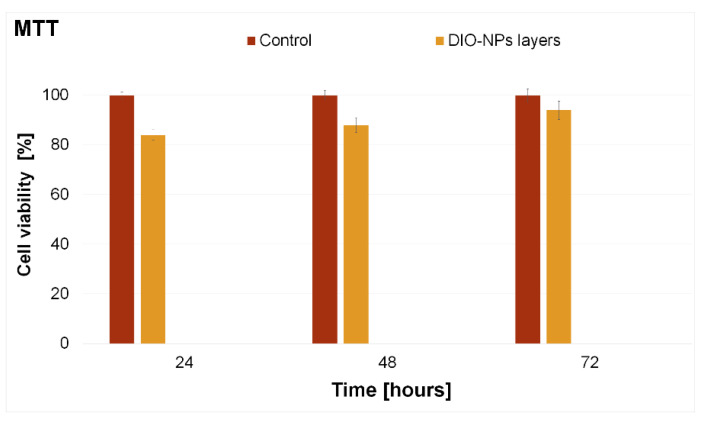
MTT assay of the viability of HepG2 incubated with DIO-NPs layers for 24, 48, and 72 h. The results are presented as means ± standard error. The data were statistically analyzed using paired and two-sample *t*-tests for means, with *p* ≤ 0.05 accepted as statistically significant.

The cytotoxicity of the DIO-NPs layers was investigated in vitro using a HepG2 cell line. The in vitro assays were performed in triplicate and the cell viability of the HepG2 cells incubated with DIO-NPs layers was assessed at three different time intervals (24, 48, and 72 h). The results of the MTT assay, which depicts the HepG2 cell viability after being incubated for different time intervals with DIO-NPs layers, are presented in Figure 9. In addition, a HepG2 cell culture was grown without being incubated with DIO-NPs layers and used as a control. The results of the MTT studies have proven that the HepG2 cells incubated with DIO-NPs layers exhibited a good viability compared to the control for all tested time intervals. More than that, the results of the MTT assay showed that, after 24 h of incubation with the DIO-NPs layers, the HepG2 viability was above 80% with a value of 84% and had an increasing tendency with the increase of the incubation time, reaching a viability of 94% after 72 h of incubation. The results of the in vitro assay highlighted that the cell viability of the HepG2 increased with the incubation time. The experiments were performed in triplicate, and the results data were presented as mean ± SD. More than that, a statistical analysis was performed using a *t*-test, and all the *p* calculated values were *p* < 0.05.

**Figure 10:**
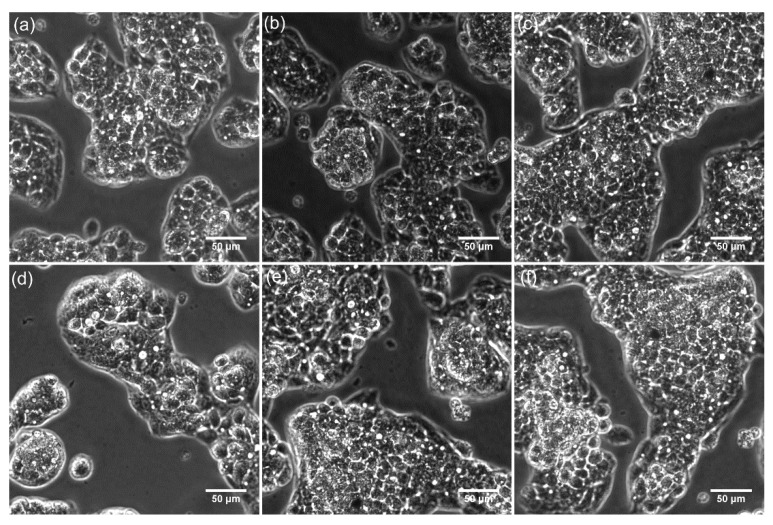
Microscopic images of HepG2 control cells (**a**–**c**) at different time intervals (24, 48, and 72 h) and HepG2 cells grown on dextran thin films deposited on glass substrate (**d**–**f**) at different time intervals (24, 48, and 72 h).

Furthermore, the HepG2 cell morphology and their adhesion and proliferation on the DIO-NPs layers surface were investigated by microscopic visualization. The 2D images of the HepG2 cells incubated on the surface of DIO-NPs layers at three different time intervals are depicted in Figure 10. The 2D images of the HepG2 cells incubated with DIO-NPs layers highlighted that the layers did not exhibit any toxic effect towards the cells for the tested time intervals. Moreover, the microscopic evaluation showed that the evaluated layers did not induce any notable changes in the morphology of the HepG2 cells compared to the control cell culture.

**Figure 11:**
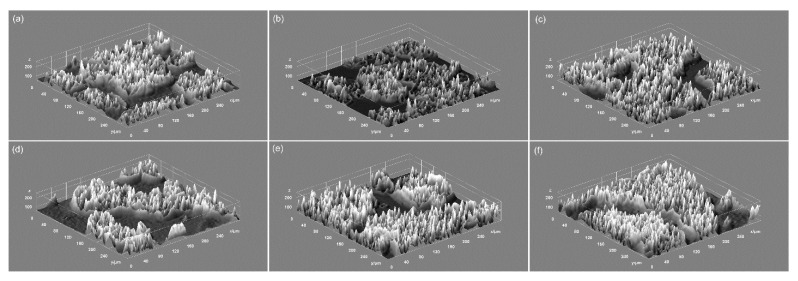
3D representation of microscopic images of HepG2 control cells (**a**–**c**) at different time intervals (24, 48, and 72 h) and HepG2 cells grown on dextran thin films deposited on glass substrate (**d**–**f**) at different time intervals (24, 48, and 72 h).

In addition, the 3D representations of the microscopic images of the HepG2 cells adhered on the surface of DIO-NPs layers were obtained using ImageJ software [40] and are depicted in Figure 11. The microscopic visualization and the 3D representation of the microscopic images have proven that the presence of DIO-NPs layers did not affect the morphology of the HepG2 cells or their proliferation rate. The microscopic images depicted the presence of a typical epithelial-like morphology with sizes of approximately 12.35 µm for the HepG2 cells incubated with the DIO-NPs for all the tested time intervals (24, 48, and 72 h).

The observations obtained by microscopic visualization are in agreement with the MTT results and highlighted that the investigated DIO-NPs layers present a good biocompatibility towards HepG2 cells and could be further investigated for the development of biomedical devices. The results are also in good agreement with previously reported data regarding the biocompatibility of layers based on iron oxide nanoparticles and iron oxide in various polymeric matrices [27,43]. The toxicity of nanocomposites and their respective coating are usually determined by a high number of factors such as nanoparticles, size, shape, porosity, surface area, wettability, incubation time, concentration, cell line, and, in the case of coatings, the substrate of the coatings as well as the roughness of the surface play an important role [53,54,55,56,57].

In the literature, the most common mechanism held accounted for the toxicity of iron oxide nanoparticles is the reactive oxygen species (ROS) formation. ROS are defined as highly reactive chemical molecules which appear due to the electron receptivity of O_2_ and are usually formed as a natural byproduct of the normal aerobic metabolism of oxygen. In the case of iron oxide nanoparticles uses in vivo, ROS have been reported to appear as by-products of the mitochondrial electron transport or to be formed via NADPH oxidase, xanthine oxidase, and nitric oxide synthase [58,59,60,61,62]. Recently, in the literature, numerous papers regarding the cytotoxicity of iron oxide nanoparticle against different cell systems were reported [63,64,65,66,67]. The data reported by these papers have evidenced that a decrease of iron oxide nanoparticle cytotoxicity was observed when dextran and other lipid coatings were employed [59,60,63,64,65,66,67,68,69,70]. In addition, these studies have emphasized that, for very low iron oxide nanoparticle concentrations, there are no significant cytotoxic effects towards the various tested cell types [63,69]. Yu et al. [59] in their study regarding the “Dextran and Polymer Polyethylene Glycol (PEG) Coating Reduce Both 5 and 30 nm Iron Oxide Nanoparticle Cytotoxicity in 2D and 3D Cell Culture” have concluded that both dextran and PEG coatings could block ROS interaction with the iron oxide nanoparticles which could lead to the prevention of the occurrence of Fenton reaction. In this case, the cell’s antioxidant defense will be capable of neutralizing ROS before being transformed into dangerous hydroxyl radicals [59]. Therefore, there are several papers reporting that a range of iron oxide nanoparticles with particular physico-chemical properties showed low or no toxicity when investigated at different concentrations and under different circumstances, either as solutions, powders, or coatings [23,24,27,43,55,69,70]. Even though each paper has a different approach of the studies, a generalization of the results is difficult, and studies have yet to be conducted in order to better understand and assess the potential toxicological risks involved in using iron oxide nanoparticle systems in biomedical applications. Nonetheless, each study makes a great contribution in the understanding of cellular toxicity mechanisms involved in the use of nanosystems in biomedical applications and expand beyond any doubt our expertise in the development of novel materials and coatings with biocompatible properties.

The previous studies reported in the literature [23,24,27,28] regarding the physico-chemical and biological properties of iron oxide/dextran thin films and suspension on glass/silicon substrate revealed that the deposition method together with their chemical composition influence the properties of the obtained layers. The study conducted by S.L. Iconaru et al. [28] reported the obtaining of magnetic iron oxide nanoparticles doped with dextran (with various concentrations of iron oxide) thin films on glass substrate by the spin coating method. SEM results highlighted that the coatings consist of regular grains with some aggregation [28]. The results of GDOES studies reveal the presence of a coating composed mainly of carbon, iron, and oxygen [28]. In addition, the results of MTT test (using hFOB 1.19 osteoblasts cell line) highlighted a good biocompatibility of the obtained layers. On the other hand, the studies conducted in preparation of iron oxide–dextran nanostructures on glass substrate by a laser technique [24,27] showed that the coatings are continuous and have a granular morphology. In addition, the results of XPS studies revealed the presence of the Fe^3+^ oxides in the samples [24,27]. The MTT assay conducted on the obtained samples using the HepG2 cell line showed their excellent biocompatibility [24,27]. The recent study, conducted by Predoi and coworkers [23], presented the results of complex research conducted on dextran-coated iron oxide nanoparticle solutions and thin films. The thin films were deposited on a Silicon substrate by the spin coating method [23]. Their results showed that the surface of the obtained thin film was uniform and homogeneous. Furthermore, the results of qualitative cytotoxicity assays (HeLa cells), revealed that dextran-coated iron oxide nanoparticle solutions and thin films did not present any toxicity after 24 and 48 h of incubation [23]. Thus, our complex study brings new information about the surface morphology of the layers, thus completing the existing results in the literature, emphasizing once again the biocompatibility of iron–dextran oxide layers. Moreover, the results obtained by optical and metallographic microscopy are presented for the first time, which emphasized the homogeneity and continuity of the layers. In addition, the results of biocompatibility studies (using HepG2 cell line) performed on iron oxide–dextran layers deposited on glass substrate by the spin coating method are presented for the first time.

## 4. Conclusions

The main purpose of the present research was to obtain iron oxide–dextran (DIO-NPs) thin layers on a glass substrate by an adapted method of synthesis. The SEM results revealed that surface morphology of the samples consists of a cobblestone-like structure. Moreover, the homogeneity and continuity of the DIO-NPs layers were highlighted both by the results obtained by AFM and by those obtained by optical and metallographic microscopy. FTIR and GDOES studies conducted on DIO-NPs layers confirmed the formation of the bonds between dextran and iron oxide nanoparticles. The cytotoxicity assay results highlighted that DIO-NPs layers did not exhibit any significant toxic effects toward the HEpG2 cell line for all the tested time intervals. Furthermore, the cytotoxicity assays on HepG2 cells also revealed that the thin films did not induce any significant morphological changes after 24, 48, and 72 h of incubation. Moreover, the quantitative MTT assay evidenced that the HepG2 cells incubated with DIO-NPs exhibited a high cell viability (84%) after 24 h of incubation. The results indicate the potential utilization of iron oxide–dextran (DIO-NPs) thin layers deposited on glass substrate in biomedical applications.

## Figures and Tables

**Table 1:** Grain size of DIO-NPs coatings obtained from different characterization methods.

Characterization Method	Grain Size (nm)
Optical microscopy	97.5 ± 7.8
Metallographic microscopy	72 ± 5.89
Scanning electron microscopy	25 ± 3
Atomic force microscopy	40.95 ± 4.55

**Table 2:** Roughness parameters of DIO-NPs coating’s surface obtained from different characterization methods.

Characterization Method	Optical Microscopy	Metallographic Microscopy	Scanning Electron Microscopy	Atomic Force Microscopy
Roughness Parameters				
R_a_	114.367	50.022	110.069	19.1050
R_q_	104.548	41.987	104.032	23.6738

## Data Availability

Data are available on demand from the corresponding author.
